# Occurrence of mesocarnivores in montane sky islands: How spatial and temporal overlap informs rabies management in a regional hotspot

**DOI:** 10.1371/journal.pone.0259260

**Published:** 2021-11-05

**Authors:** Amanda M. Veals, John L. Koprowski, David L. Bergman, Kurt C. VerCauteren, David B. Wester

**Affiliations:** 1 School of Natural Resources and the Environment, University of Arizona, Tucson, Arizona, United States of America; 2 United States Department of Agriculture, Animal and Plant Health Inspection Service-Wildlife Services, Phoenix, Arizona, United States of America; 3 United States Department of Agriculture, National Wildlife Research Center, Animal and Plant Health Inspection Service-Wildlife Services, Fort Collins, Colorado, United States of America; 4 Texas A&M University-Kingsville, Kingsville, Texas, United States of America; University of Oklahoma Norman Campus: The University of Oklahoma, UNITED STATES

## Abstract

Interspecific interactions among mesocarnivores can influence community dynamics and resource partitioning. Insights into these interactions can enhance understanding of local ecological processes that have impacts on pathogen transmission, such as the rabies lyssavirus. Host species ecology can provide an important baseline for disease management strategies especially in biologically diverse ecosystems and heterogeneous landscapes. We used a mesocarnivore guild native to the southwestern United States, a regional rabies hotspot, that are prone to rabies outbreaks as our study system. Gray foxes (*Urocyon cinereoargenteus*), striped skunks (*Mephitis mephitis*), bobcats (*Lynx rufus*), and coyotes (*Canis latrans*) share large portions of their geographic ranges and can compete for resources, occupy similar niches, and influence population dynamics of each other. We deployed 80 cameras across two mountain ranges in Arizona, stratified by vegetation type. We used two-stage modeling to gain insight into species occurrence and co-occurrence patterns. There was strong evidence for the effects of elevation, season, and temperature impacting detection probability of all four species, with understory height and canopy cover also influencing gray foxes and skunks. For all four mesocarnivores, a second stage multi-species co-occurrence model better explained patterns of detection than the single-species occurrence model. These four species are influencing the space use of each other and are likely competing for resources seasonally. We did not observe spatial partitioning between these competitors, likely due to an abundance of cover and food resources in the biologically diverse system we studied. From our results we can draw inferences on community dynamics to inform rabies management in a regional hotspot. Understanding environmental factors in disease hotspots can provide useful information to develop more reliable early-warning systems for viral outbreaks. We recommend that disease management focus on delivering oral vaccine baits onto the landscape when natural food resources are less abundant, specifically during the two drier seasons in Arizona (pre-monsoon spring and autumn) to maximize intake by all mesocarnivores.

## Introduction

Ecological processes across ecosystems can be shaped by species interactions [1]. Further, natural population dynamics and community structure are greatly influenced by interactions among species [2–4]. Interspecific competition and niche partitioning can be important organizing mechanisms for community diversity, especially between species with similar ecological niches [1] Interactions among species are capable of shaping individual behavior as well as population dynamics [5–7]. Interspecific interactions such as competition, aggression, and predation can impact potential coexistence between different species [8,9].

The distribution of a given species can be influenced by interspecific interactions, therefore impacting habitat use and resource acquisition [1,10,11]. Competitive exclusion or aggression between species that share similar niches can prevent coexistence [1]. Additionally, at higher trophic levels, interspecific competition can be especially important [12,13], particularly in situations where intraguild predation may occur as an extreme case of interference competition [14,15]. Interactions among carnivores have profound effects on ecosystem functioning [11] and influence populations of other carnivores and prey species [16,17]. Species may be able to mutually occur through niche segregation [1,8–9]. The ability for ≥2 species to coexist is fundamentally a function of niche overlap and therefore interspecific interactions [1,9]. Spatial coexistence between overlapping carnivores may be possible through fine-scale partitioning of resources [9,18].Examining carnivore co-occurrence patterns can help identify the underlying factors affecting local species distributions, ecological functions, and partitioning of resources [8,9,19].

Spatial overlap between species, particularly those competing for similar resources, has important potential for pathogen spillover [20]. Knowledge of wildlife spatial ecology can inform disease epidemiology and potential spread of zoonotic diseases [21]. Epizootiology, the study of disease patterns within wildlife populations, for many wildlife diseases can be influenced by habitat requirements of host/reservoir species. By examining habitat use of a host/reservoir species for zoonotic diseases, management strategies can be improved [22]. Further, understanding the impact interspecific species have on habitat use and occurrence patterns of a host species can improve our understanding of potential pathogen transmission [20]. Our need to understand zoonotic diseases and the pathways of transmission has been highlighted with the emergence of novel zoonoses, including COVID-19. Understanding the environmental factors that influence viral spread and other risk factors can improve capabilities to predict pandemics and prepare for the next resurgence [23].

Widespread and far ranging mesocarnivores can often be reservoirs for prevalent zoonotic diseases, which make this group of organisms useful models for understanding how species’ spatial distribution can inform wildlife disease management [24]. Mesocarnivores are the primary rabies reservoirs in the United States [25,26]. Mesocarnivores, particularly striped skunks (*Mephitis mephitis*) and gray foxes (*Urocyon cinereoargenteus*), play a major role in rabies outbreaks throughout the US [27,28]. Striped skunks are the primary reservoir for the south-central rabies variant and gray foxes are the primary reservoir for two unique variants in the US and Mexico [27,29,30]. Understanding the role mesocarnivores play in rabies outbreaks in a regional hotspot is critical for disease management and nationwide efforts to eliminate rabies in wild carnivores [27].

A regional rabies hotspot, located in the southwestern US, has a large, intact mesocarnivore guild, making it an ideal location to examine interspecific interactions and draw conclusions about community dynamics. We examined co-occurrence patterns of the four most abundant mesocarnivores that have overlapping geographic distributions in this region: gray foxes, striped skunks, bobcats (*Lynx rufus*), and coyotes (*Canis latrans*). Additionally, these species can compete for resources, occupy similar niches, and influence population dynamics of each other across their geographic ranges [13,15,31–35], all of which can influence transmission risk and therefore disease management.

We conducted our study in two mountain ranges within the Madrean Archipelago ecosystem of Arizona. This ecosystem has high levels of biological diversity [36] and is located within the regional rabies hotspot. Host species ecology can provide an important baseline for disease management strategies [21,24], especially in biologically diverse ecosystems and heterogeneous landscapes. In this region, we hypothesized the spatial ecology of mesocarnivores is greatly influenced by interspecific interactions. We used a mesocarnivore guild native to this region that is prone to rabies outbreaks as our study system. The goals of this study were to 1) examine multi-season occurrence patterns of mesocarnivores across vegetation communities, 2) understand how overlap with other mesocarnivores influences occurrence, and 3) draw inferences on community dynamics to inform rabies management in a US rabies hotspot. We hypothesized season and vegetation community would influence occurrence of species due to availability of cover and food resources. We predicted co-occurrence patterns would be influenced by competition for food resources and risk of intraguild predation. The presence of species with larger body sizes (i.e., coyotes and bobcats) would have negative impacts on smaller species (i.e., foxes and skunks). Additionally, we hypothesized that species with generalist diets (i.e., foxes, skunks, and coyotes) would be less likely to co-occur with each other. Our goal was to evaluate how heterogeneity in available vegetation communities within our study areas could influence mesocarnivore occurrence and spatial overlap [37,38]. This study quantifies interspecific interactions across space to draw inferences for disease management.

## Materials and methods

### Study area

We selected the Pinaleño Mountains (32.70163°N, -109.87189°E) and the White Mountains (33.78512°N, -109.24785°E) in southeastern Arizona for comparison due to their similarity in overall elevation and vegetation communities [31,39,40]. The Pinaleño Mountains comprise 780 km^2^ of forested area and are a part of the Madrean Archipelago [41]. The White Mountains are approximately 13,000 km^2^ of continuous forest. Within the upper elevations the vegetation communities across both mountain ranges are composed of cork-bark fir (*Abies lasiocarpa* var. *arizonica*), Douglas fir (*Pseudotsuga menziesii*), Engelmann spruce (*Picea engelmanii*), ponderosa pine (*Pinus ponderosa*), quaking aspen (*Populus tremuloides*), southwestern white pine (*Pinus strobiformis*) and white fir (*Abies concolor*) [39,42].

### Camera trapping

**Fig 1:**
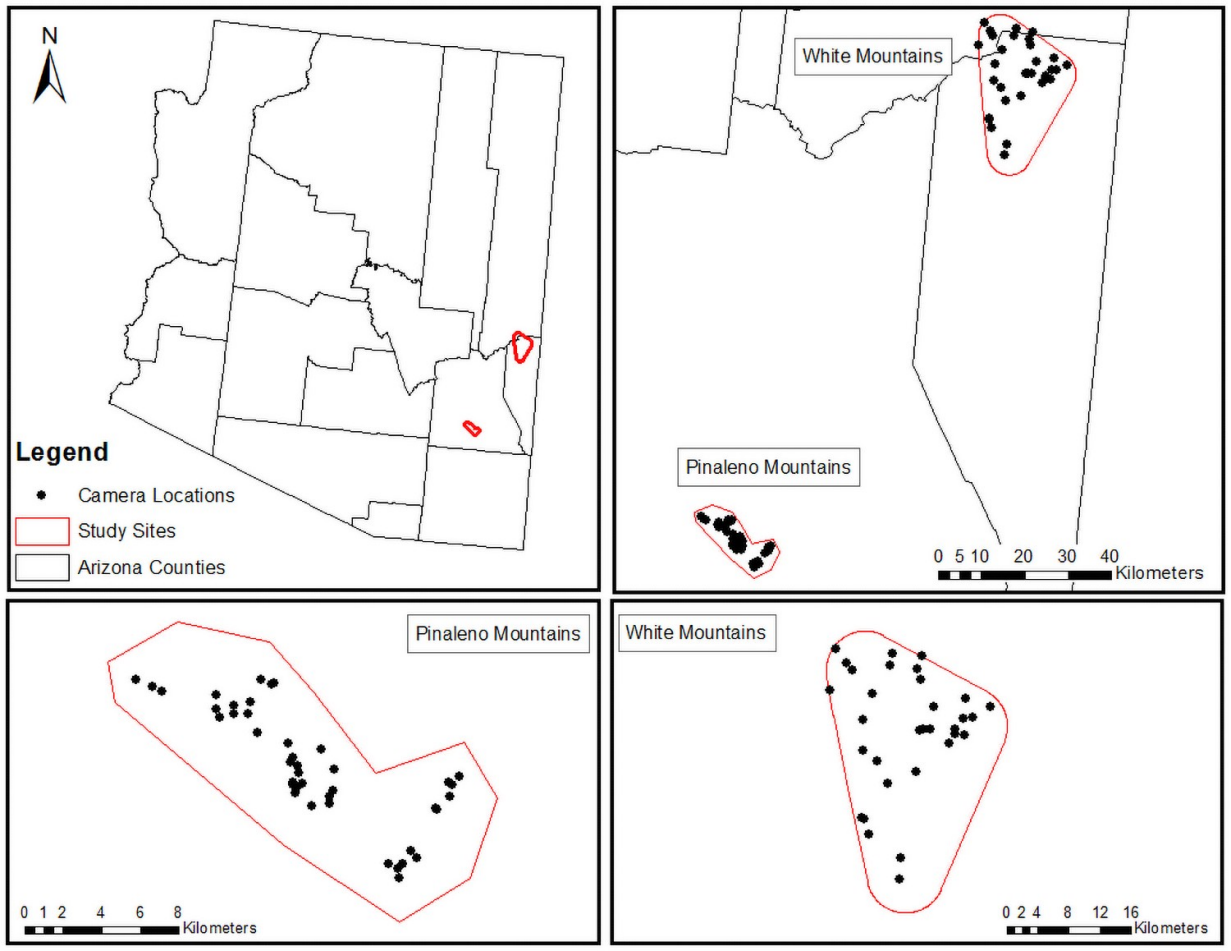
Map of study sites and camera trap array in southeastern Arizona.

We deployed camera traps in the Pinaleño and White Mountains: one array of 40 camera traps in each study site active between June 2016 and August 2017 (Fig 1). We kept cameras active in each location for approximately 1 year to collect multi-seasonal data across annual weather patterns. Each array was a stratified random sample by available vegetation communities with a minimum distance of 1 km between cameras. We used Geographic Information Systems (GIS, ESRI, Redlands, CA) to randomize camera locations across vegetation communities. Vegetation communities were classified as polygons and defined by the species or genera of greatest abundance, based on the uppermost canopy of the plant community [44,45]. Cameras were placed at an average elevation of 2542 m ± 325.7 (mean ± SD; range = 1531–3037 m). We used HD Trophy digital cameras (Bushnell Inc., Overland Park, KS) which were protected in an iron box, locked with a cable and affixed to a suitable tree within 5 m of the random location and approximately 50 cm from the ground. Cameras were set to take a single photo when triggered by motion with no delay between successive trigger events. No lure or bait was used. Cameras were checked every six weeks for camera function, battery charge, and data download. A species was considered detected if it was photographed at least once during a survey (one Julian day). Detection was considered a binary response variable such that all photographs were condensed into one detection per day for a given species.

All field work was conducted in accordance with the American Society of Mammalogists guidelines [43] and approved by the University of Arizona’s Institutional Animal Care and Use Committee (IACUC protocol #14–504). Research was conducted under Wildlife Services authority as outlined in the Act of March 2, 1931, (46 Stat. 1468; 7 USC 426-426b).

### Environmental variables

We placed a 10 m-radius vegetation plot centered around each camera. We surveyed vegetation communities present across both mountain ranges [44,45], which we grouped into four categories and classified the surrounding vegetation as: upper evergreen forest, upper pine-oak woodlands, ponderosa pine forests, and pine-oak-juniper woodlands. Understory and overstory measurements were completed in all four cardinal directions at 5 m and 10 m out from the base of the tree bearing the camera. We used the Strickler method to estimate canopy cover [46]. A cover pole measuring 2.5 cm × 200 cm marked every 10 cm was used to measure understory density [47] and we noted any obscuring vegetation taller than the pole as > 2 m. We compared vegetation structure between the two study sites using a t-test (P < 0.05) and examined the vegetation community composition.

We identified six seasons as sampling periods, which we classified by monthly precipitation and temperature for 2016–2017. We classified January, February, and March as winter, a cold and wet season; April, May, and June as pre-monsoon spring, a relatively hot and dry season; July, August, and September as monsoon summer, a hot and wet season; and October, November, and December as autumn, a relatively cold and dry season [48]. Our six seasons included: spring 1, summer 1, autumn, winter, spring 2, and summer 2.

Vegetation characteristics (canopy cover, understory height, vegetation community type), elevation, study site, season, and daily average temperature were used as model variables to evaluate potential effects on occurrence. Canopy cover and understory height were sampled at each camera location, while remaining variables were determined from GIS layers [44,45] and local weather sampling stations [49,50]. We found an interaction between season and vegetation community; when a model included both season and vegetation community, we also included the interaction term. We used spring 2 as our reference category for season and upper pine-oak forest as our reference category for vegetation community categorical variables. Climate and resource availability can change drastically across seasons especially in areas with large elevation gradients [51] like our study sites. We felt these variables captured microclimatic differences at the camera-site scale as well as seasonal variation.

### Single-species occurrence

We used two-stage modeling to gain insight into species occurrence and co-occurrence patterns to make inferences for pathogen transmission and management. Stage one of the modeling process used generalized linear models to identify factors that explained variation in occurrence (i.e., detection probability) of each mesocarnivore species. We determined models *a priori* based on mesocarnivore ecology [51]. We predicted environmental factors would impact availability of cover and food resources, and therefore influence mesocarnivore occurrence at sites.

We used a multiple logistic regression to estimate probability of mesocarnivore occurrence at sites using the GLIMMIX and MIXED procedures (SAS/STAT software Version 9.4, Cary, NC) for generalized linear mixed and linear mixed models respectively. We ran four sets of models, with the detection probability of a mesocarnivore species (gray fox, skunk, coyote, or bobcat) as the response variable. For each species, we tested a set of *a priori* models that represented biological hypotheses (Table 1). Selection among candidate models that differ in their fixed effects is not appropriate with this method because it bases its default estimation on a residual likelihood. Thus to compare candidate models for each species, we followed Ten Eyck and Cavanaugh [52] by (1) analyzing a full model with GLIMMIX (with all possible subsets of variables) to generate pseudo-data which were then (2) analyzed by the MIXED procedure to fit all candidate models of interest and estimate information criteria suitable for model selection purposes (the best fit model(s) were based on ΔAICc scores of > 2); the selected models were (3) analyzed by GLIMMIX to estimate regression coefficients of interest.

**Table 1:** Candidate models for single-species occurrence based on *a priori* hypotheses.

Model	Variables
1	Vegetation Type + Elevation + Season + Season×Vegetation Type + Temperature
2	Vegetation Type + Canopy Cover + Understory + Season + Season×Vegetation Type + Temperature
3	Vegetation Type + Season + Season×Vegetation Type + Temperature
4	Elevation + Season + Temperature
5	Elevation + Canopy Cover + Understory + Season + Temperature
6	Canopy Cover + Understory + Season + Temperature

The same candidate models were applied to all four mesocarnivores with probability of detection as the response variable.

For generalized linear models, pseudo-likelihood methods are conventional. In this model-fitting approach, pseudo-data (which are approximately normally distributed) are generated from the response variable via a Taylor-series expansion based both on the model and the response variable. Because pseudo-data depend, in part, on the model, they are not consistent for different models, and, because the resulting pseudo-likelihoods that are not comparable, neither are selection criteria based on them comparable. Our approach follows Ten Eyck and Cavanaugh [52] in that PROC GLIMMIX was used to generate pseudo-data, which were then analyzed with PROC MIXED with maximum likelihood methods whose AICc values are comparable.

### Multi-species co-occurrence

For the second stage of modeling, we examined how probability of detecting a species at a site was influenced by the probability of detecting one or more other mesocarnivore species at the site (Table 2). For example, we modeled probability of gray fox occurrence as a function of environmental variables and the probability of detecting coyotes, bobcats, and/or skunks at a site. We estimated season- and camera-specific probabilities of detection for each of the four mesocarnivores by regressing incidence on camera for each season; estimated probabilities were equal to the observed frequency of occurrence. Our candidate multi-species occurrence models were based on the probability of detection from the top single-species occurrence model (i.e., stage 1; Table 1). We compared this baseline occurrence model to several models that included camera-season specific probabilities of detection for other mesocarnivores (Table 2). If there were competing models in stage 1, we included both models in stage 2 to compare.

**Table 2:** Candidate models for multi-species occurrence.

Model	Variables
1	Stage 1
2	Stage 1 + Mesocarnivore 1
3	Stage 1 + Mesocarnivore 2
4	Stage 1 + Mesocarnivore 3
5	Stage 1 + Mesocarnivore 1 + Mesocarnivore 2
6	Stage 1 + Mesocarnivore 1 + Mesocarnivore 3
7	Stage 1 + Mesocarnivore 2 + Mesocarnivore 3
8	Stage 1 + Mesocarnivore 1 + Mesocarnivore 2 + Mesocarnivore 3

Stage 1 represents the top model from the single-species occurrence modeling exercise. We then compared that model to a set of new models that included the probability of detecting another mesocarnivore species at a site. The response variable was the probability of detecting the target carnivore species (e.g., gray fox), Mesocarnivore 1–3 represents the probability of detecting one of the other three carnivore species at that site (e.g., Mesocarnivore 1 = skunk probability).

We used a multiple logistic regression using the GLIMMIX and MIXED procedures (as described above) to estimate probability of mesocarnivore occurrence at sites as a function of environmental variables and the probability that other mesocarnivore species were present at that site. For models to be compared, we had to subset the response data, detection of a species, to observations for a site that had a corresponding probability of detection for at least one of the other three mesocarnivores.

## Results

We surveyed 80 sites for 446 days between 8 June 2016 and 27 August 2018 for a total of 35,680 trap days. Gray foxes, bobcats, and skunks were detected across the entire range of elevations that we surveyed (1,530–2,984 m). Coyotes were detected across the entire range of elevations surveyed in the White Mountains (2,180–2,805 m). Gray foxes, bobcats, and skunks were detected across all available vegetation communities. Coyotes were not detected in upper pine-oak however, this vegetation community was truly only available in the Pinaleño Mountains where the species was never detected by our cameras (Table 3).

**Table 3:** Species detections across sampling sites.

	Pinaleño Mountains				White Mountains			
	Pine-oak-juniper woodlands	Ponderosa pine forest	Upper evergreen forest	Upper pine-oak woodlands	Pine-oak-juniper woodlands	Ponderosa pine forest	Upper evergreen forest	Upper pine-oak woodlands
Total Camera Days	1,534	3,885	226	1,307	1,292	2,115	4,006	9
Fox Detections	21	84	25	143	18	47	182	0
Skunk Detections	60	131	111	87	12	9	110	0
Bobcat Detections	11	55	37	5	2	3	21	0
Coyote Detections	0	0	0	0	17	70	111	0

The Pinaleño Mountains were dominated by upper evergreen forest (62.5%) whereas the White Mountains were more heterogenous, composed of ponderosa pine woodlands (42.5%), upper evergreen forest (30.0%), and pine-oak-juniper woodlands (25.0%). Vegetation and structural characteristics varied between our two study sites. Canopy cover was higher in the Pinaleño Mountains (64.1 ± 15.9% [SD]) than the White Mountains (53.5 ± 23.9%; t_50.37_ = 1.012, *p* = 0.032), whereas understory height did not differ between the Pinaleño Mountains (106.9 ± 85.2 cm) and the White Mountains (94.5 ± 89.4 cm; t_78.61_ = -0.637, *p* = 0.526).

### Single-species occurrence

For stage one, modeling single-species occurrence patterns, we found differences in the environmental variables that influenced probability of detection for the four species (S1–S3 Tables). Gray fox occurrence was best explained by a model that included elevation, season, temperature, canopy cover, and understory height (S1 Table). In this model, occurrence was negatively influenced by elevation while temperature, canopy cover, and understory height had a positive relationship. Gray foxes were more likely to occur in the summer 1, autumn, and winter seasons (S2 Table).

The best fit model for skunks included elevation, season, temperature, canopy cover, and understory height (S1 Table), the same variables that influenced foxes. In this model, skunk occurrence was positively influenced by temperature, canopy cover, and understory height but negatively associated with elevation. Occurrence was higher in all seasons compared to the reference season of pre-monsoon spring 2 (S2 Table).

A model that included vegetation community, elevation, season, season×vegetation community interaction, and temperature best explained bobcat occurrence (S1 Table). In this model, bobcats were more likely to occur in all other vegetation communities compared to upper pine-oak forest. Occurrence was higher in the summer in pine-oak-juniper woodlands and ponderosa pine forests. Our top model also included elevation ($\hat{\beta }=0.23\pm 0.04$, *p* < 0.0001) and temperature ($\hat{\beta }=0.02\pm 0.02$, *p* > 0.25; S2 Table).

Coyotes had two competing models based on ΔAICc scores (S1 Table). The best fit model (w = 0.526) included vegetation community, elevation, season, season×vegetation community interaction, and temperature, with the competing model being very similar but without including elevation. Occurrence was negatively influenced by elevation and temperature in the top model. Coyotes were less likely to occur in ponderosa pine forests during the summer 1 and winter seasons as well as in pine-oak-juniper woodlands in winter (S2 Table).

### Multi-species co-occurrence

**Fig 2:**
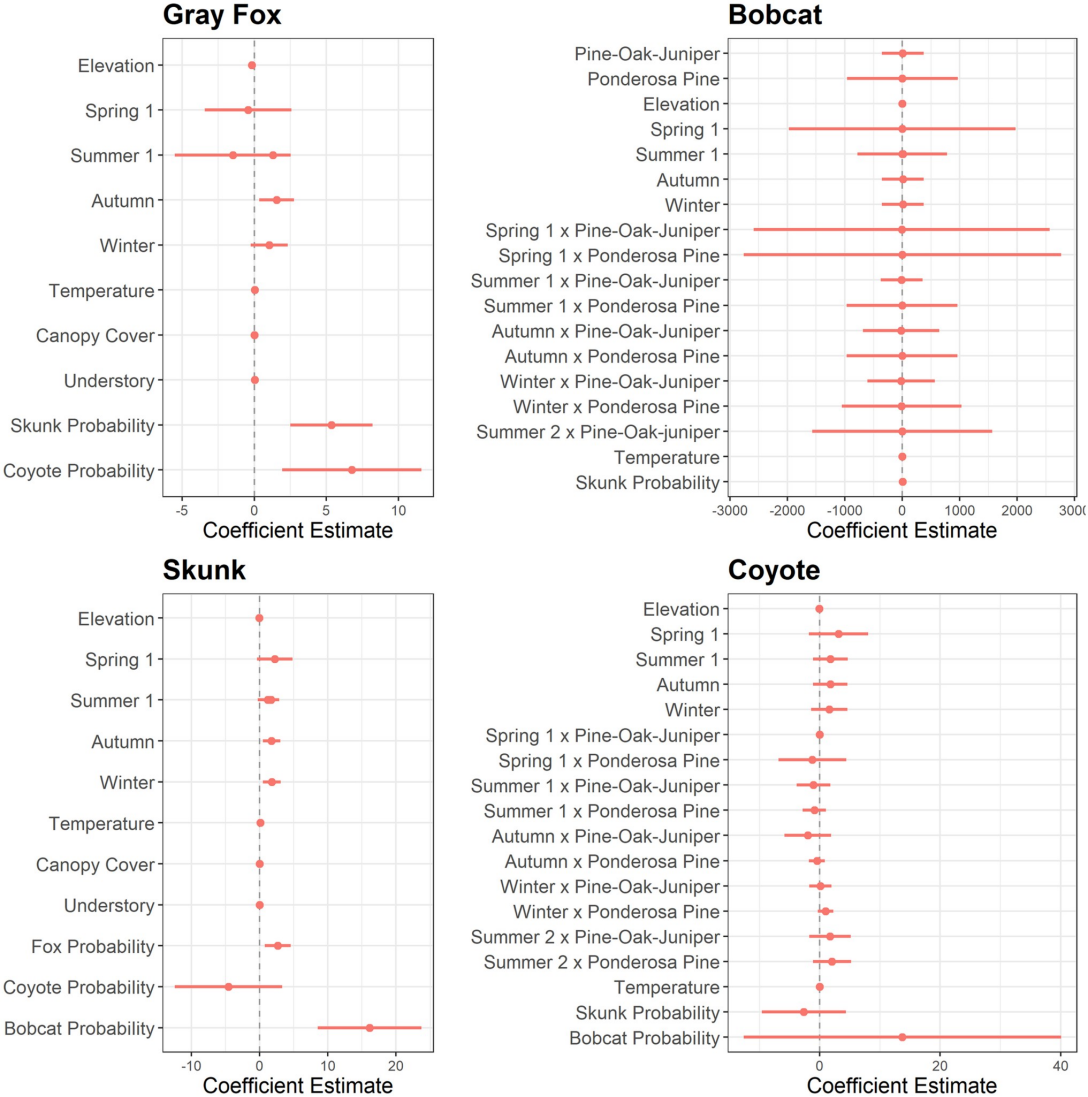
Beta estimates for best fit model from stage 2 (multi-species co-occurrence) for each mesocarnivore. We compared the beta estimate for each variable to zero with a 95% confidence interval to determine if the variable positively or negatively influenced probability of detection. The x-axis depicts beta estimates for each coefficient and the y-axis shows all coefficients included in the best fit stage 2 model for each mesocarnivore (gray fox, skunk, bobcat, coyote).

We compared probability of mesocarnivore occurrence as a function of environmental variation in the presence of other potential competitors. We compared the best fit model from stage 1 as a pseudo-null model for each mesocarnivore to models that included camera-season specific probability of detection for one or more other mesocarnivore (Table 2). We then plotted beta estimates for this best fit stage 2 model to examine the effect environmental variables and the presence of competing carnivores had on the detection probability of a target species (Fig 2). Gray fox co-occurrence in stage 2 had two top competing models based on Δ AICc scores (S1 Table). Our best fit model (w = 0.722) indicated gray fox co-occurrence was positively influenced by coyote and skunk detection at a site (Tables 4 and S2). We found that skunk co-occurrence was positively associated with gray fox and bobcat detection but negatively influenced by coyote detection (Tables 4 and S2). Bobcat co-occurrence in stage 2 had three competing models based on Δ AICc scores (S1 Table). Our best fit model (w = 0.473) indicated that bobcat co-occurrence was significantly positively associated with skunk detection (Tables 4 and S2).

**Table 4:** Beta estimates for multi-species co-occurrence from stage 2.

	Focal Species			
Influential Species	Fox	Skunk	Bobcat	Coyote
Fox	-	2.642 ± 0.494*	NA	NA
Skunk	5.356 ± 0.741*	-	6.098 ± 1.598	-2.653 ± 1.827
Bobcat	NA	16.095 ± 1.979*	-	13.716 ± 6.897*
Coyote	6.754 ± 1.256*	-4.557 ± 2.053*	NA	-

Coyote occurrence in stage 1 had two competing models; therefore, we included both as pseudo-null models in stage 2. In the second stage, there were nine competing models based on Δ AICc scores (S1 Table). Across these top models, we found trends in significant effects (P < 0.10) [53] and their influence on coyote co-occurrence (S3 Table). All nine models had significant effect for season as well as season×vegetation type. Three models included significant positive effect for bobcat probability. An additional three models included bobcat probability in the model, but the coefficient was not significant (Table 4).

## Discussion

Mesocarnivores demonstrated only modest separation across landscapes. Gray foxes, striped skunks, bobcats, and coyotes are considered habitat generalists across their geographic range [31,40,54–56]. These species can compete for resources, occupy similar niches, and influence population dynamics of each other [13,15,32–35,57]. We observed patterns of occurrence and co-occurrence that are consistent with these previous findings. We found evidence of mutual coexistence among these species, likely through fine scale niche portioning.

Gray foxes and skunks used a wide variety of vegetation communities but were detected more in sites with higher canopy and understory cover. Additionally, foxes and skunks used sites at lower elevations with warmer average daily temperatures. Foxes and skunks are generalists with an omnivorous diet [40,55], which could explain these species occurring at lower elevations to seek out alternate food sources across seasons. Seasonal distributions of omnivores and mesocarnivores likely reflect temporal variation in a wide range of food resources such as fruits, nuts, insects, small vertebrates, and bird eggs [48,58].

Bobcats were also detected at sites with warmer temperatures, however used sites at higher elevations. Canopy cover and understory height did not impact bobcat occurrence, but vegetation community did. Bobcats were positively associated with pine-oak-juniper woodlands and ponderosa pine forests, especially in the hot and wet monsoon summer season. Summer in Arizona is likely when females are raising kittens with resulting changes in seasonal habitat use possible [31,56]. Bobcats are considered habitat generalists as well, however, as strict carnivores they have a more specific diet than foxes or skunks [56,59,60]. Occurrence patterns of bobcats could be related to prey availability and cover [9], which we did not measure directly.

Coyote occurrence was complicated by the lack of detections in the Pinaleño Mountains. Vegetation community, season, and the interaction between these two variables impacted coyote detection. Coyotes were negatively associated with ponderosa pine forests in both summer 1 and winter, as well as pine-oak-juniper woodlands in winter. As another habitat generalist with an omnivorous diet, coyotes are flexible in their habitat use [54,60]. Differences in coyote occurrence in summer 1 and summer 2 are likely due to sampling. Our cameras were active all three months of summer 1, but due to sampling constraints, cameras were only active through August of summer 2.

Intraguild associations affected occurrence across landscapes. For all four mesocarnivores, a multi-species co-occurrence model better explained patterns of detection than the single-species occurrence model. Gray foxes were more likely to occur in areas where skunks and coyotes were also detected. Foxes and skunks as generalist omnivores, can likely co-occur without much competition [18]. However, the relationship with fox occurrence and coyote detection probability is more complicated. Coyotes have been documented to negatively impact gray fox space use [57], with gray foxes maintaining core areas that did not overlap substantially with those of coyotes or bobcats [32], suggesting avoidance of areas of high use by the larger carnivores [34]. Gray foxes are sympatric with coyotes throughout much of North America and coyotes have been documented to depredate upon foxes [15]. It is likely that we are not documenting potential spatial partitioning expected by foxes to avoid conflict with coyotes at the spatial scale of our cameras.

Skunk occurrence was positively influenced by fox and bobcat presence, but negatively by coyotes. Bobcats were positively associated with skunk detection. Skunks are omnivorous and bobcats are strictly carnivorous [59], indicating there is no direct competition for food resources. Coexistence between these two species is likely facilitated by niche partitioning and differences in body size [8,61]. Skunks were less likely to be detected at sites where coyotes were also detected, which followed our hypothesis. Coyote-skunk interactions have not been thoroughly examined, however, similar to spatial avoidance shown by other smaller mesocarnivores to coyotes, it is likely we are detecting spatial partitioning by skunks to avoid conflict with the larger coyotes [7,32].

Coyotes had nine competing multi-species co-occurrence models, making the interpretation of our results complex. However, we noted trends across these competing models with a positive effect for bobcat probability across six of these top models. In many studies within the US, bobcats and coyotes often shared space with neither species exhibiting spatial or temporal partitioning [33,62–64]. In south Texas, presence of coyotes was a positive indicator of bobcat occurrence [9], where the positive effects were likely due to an abundance of preferred cover and high availability of food. Competition may be reduced between coyotes and bobcats occurring in areas with moderate prey populations or greater variety of food items for coyotes [65].

Thornton et al. [62] suggested that reduced agonistic encounters between species might be attributed to non-overlapping core areas, even in areas where the species do not segregate at the home range scale. The Madrean Archipelago is a biologically diverse ecosystem [36,66], likely offering an abundance of cover and food resources for these mesocarnivores. These four species are influencing the space use of each other in complex ways, potentially as a function of variables we did not measure such as availability of food resources or cover, and human disturbance such as roads. We documented overlap in site use by the four species, and patterns of co-occurrence which are likely best explained by space and resource partitioning. However, it is possible that direct and indirect human presence on the landscape could be influencing occurrence patterns for these mesocarnivores [9,60], which would have implications for disease management.

We documented co-occurrence as a mechanism to understand potential for interactions among rabies reservoirs. Rabies is a virulent disease that can easily spillover from one mammalian species into another [29], making epizootic events difficult to manage. Gray foxes and striped skunks are two of the largest reservoirs for rabies in Arizona and many recent outbreaks have occurred in both species [67]. Rabies positive coyotes and bobcats seem to be less common in Arizona [28], which could be a function of lower population densities and social structure. These four mesocarnivore species have been documented to tolerate human disturbance and use urban areas [60,68,69], and as habitat generalists that can exist close to humans, these species have the potential to interact with not just each other, but also domestic animals and people.

Spatial overlap between species that occupy similar niches can influence pathogen spillover and transmission rates [20]. We found similarities in occurrence patterns by gray foxes, skunks, coyotes, and bobcats in a regional rabies hotspot. Further, our results show presence of intraguild species affected co-occurrence across landscapes. Knowledge of wildlife spatial ecology can inform disease epidemiology and potential spread of zoonotic diseases like rabies [20]. Surveillance of viruses in the environment and in hotspots can provide useful information to predict and quell outbreaks of zoonotic diseases [23].

## Conclusions

Oral rabies vaccination (ORV) and trap-vaccinate-release programs have been successful in combating rabies in mesocarnivores across urban and rural areas [70]. These areas where mesocarnivores co-occur should be considered in both proactive and reactive strategies for controlling the spread of rabies in Arizona. Diet overlap among carnivores could impact uptake rates of oral vaccines by specific species [71,72], but since all are susceptible to rabies this is a positive outcome. We recommend that rabies managers deliver oral vaccine baits onto the landscape when natural food resources are most scarce, particularly in the two drier seasons in Arizona (pre-monsoon spring and autumn). Baiting multiple times per year with a focus on these two seasons when mesocarnivores are most active on the landscape [73,74] could optimize the number of individuals of all species that consume ORV baits. We observed higher detection probabilities in both stage 1 and stage 2 models for all four mesocarnivore species in autumn and some species in the pre-monsoon spring. This could indicate that animals are more active and searching for resources. Competition for resources is most likely higher during these times of year, however, animals may be more likely to find and eat baits in times of low food availability.

The pre-monsoon spring season coincides with mating and denning periods for these mesocarnivores [31,40,54–56]. Increased intraspecific interactions during mating and denning can influence pathogen transmission. Additionally, it has been documented that rabies can be transmitted vertically from mother to litter [75], which could preserve the virus in wildlife reservoirs. Pre-monsoon spring is a key season for disease management as adult animals are likely more susceptible due to increased contact rates from breeding and young of the year are susceptible from vertical transmission.

Distribution across space and time plays a role in intra- and interspecific interactions and therefore disease spread. Understanding what vegetation communities, elevations, and general landscape features that are more likely to be occupied by rabies vector species at certain times of the year, can increase efficiency and minimize costs for disease management techniques. Rabies management strategies that considers spatial overlap and resource competition among rabies reservoirs will likely have a higher success rate for protecting wildlife populations, domestic animals, and humans [76–78].

Mesocarnivore co-occurrence in southeastern Arizona has important implications for disease management. Direct and indirect pathogen transmission risk can be assessed from host species overlapping in space use across time. We observed that these mesocarnivores are co-occurring across elevation bands, vegetation communities, and seasons. Disease management in our study area would benefit from more targeted research on overlap between mesocarnivores in space and time in southeastern Arizona. Our results lay the groundwork for future studies focused on interspecific interactions, niche partitioning, and pathogen transmission. A multi-scale habitat use analysis focused on species interactions would add to our understanding of how mesocarnivores are co-occurring in local sites.

## Supporting information

S1 Table Describes model rank and weights for each mesocarnivore species for Stage 1 (single-species occurrence) and Stage 2 (multi-species occurrence). (DOCX)

S2 Table Describes model outputs for best fit models for each mesocarnivore for Stage 1 and Stage 2. (DOCX)

S3 Table Describes the Type 3 test of fixed effects for two stage modeling process for each mesocarnivore. (DOCX)

S1 File Camera specific detections. Raw data file with an excel sheet page that contains the 0/1 detection data for each mesocarnivore species (gray fox, skunk, bobcat, and coyote). Column “Camera” represents the unique camera ID (1–63), “Season” represents sampling season (1–6), “Day” represents the day within each season (unique Julian date, 1–92), and “Detection” represents if a photo was taken of the target species (0 = no detection, 1 = detection, NA = camera not operational).
(XLSX)
