# Supplementary material for: Occurrence of mesocarnivores in montane sky islands: How spatial and temporal overlap informs rabies management in a regional hotspot
Source: PLoS One. 2021 Nov 5;16(11):e0259260. doi: 10.1371/journal.pone.0259260 (PMC8570508; doi:10.1371/journal.pone.0259260)
Supplement: S1 Table — (DOCX) [file pone.0259260.s001.docx]

**S1 Table.** **Model rank and weights.** Models ranked by Δ AICc and weight (*w*) for each mesocarnivore species for Stage 1 and Stage 2. Stage 1 = single-species occurrence, Stage 2 = multi-species occurrence. A = top model, B = competing model.

*Gray Fox*

| **Stage 1 Models** | **Δ AICc** | ***w*** |
| --- | --- | --- |
| Elevation + Canopy Cover + Understory + Season + Temperature | 0.000 | 0.737 |
| Vegetation Type + Elevation + Season + Season*Vegetation Type + Temperature | 2.062 | 0.263 |
| Elevation + Season + Temperature | 75.356 | 0.000 |
| Vegetation Type + Canopy Cover + Understory + Season + Season* Vegetation Type + Temperature | 173.710 | 0.000 |
| Vegetation Type + Season + Season*Vegetation Type + Temperature | 212.535 | 0.000 |
| Canopy Cover + Understory + Season + Temperature | 242.266 | 0.000 |
| **Stage 2 Models** | **Δ AICc** | ***w*** |
| Stage 1 + Skunk Probability + Coyote Probability | 0.000 | 0.722 |
| Stage 1 + Skunk Probability + Coyote Probability + Bobcat Probability | 1.995 | 0.266 |
| Stage 1 + Skunk Probability | 10.165 | 0.004 |
| Stage 1 + Coyote Probability | 10.575 | 0.004 |
| Stage 1 + Coyote Probability + Bobcat Probability | 11.905 | 0.002 |
| Stage 1 + Skunk Probability + Bobcat Probability | 12.169 | 0.002 |
| Stage 1 | 18.774 | 0.000 |
| Stage 1 + Bobcat Probability | 20.381 | 0.000 |

*Skunk*

| **Stage 1 Models** | **Δ AICc** | ***w*** |
| --- | --- | --- |
| Elevation + Canopy Cover + Understory + Season + Temperature | 0.000 | 0.996 |
| Vegetation Type + Canopy Cover + Understory + Season + Season*Vegetation Type + Temperature | 11.167 | 0.004 |
| Canopy Cover + Understory + Season + Temperature | 21.128 | 0.000 |
| Vegetation Type + Elevation + Season + Season*Vegetation Type + Temperature | 47.126 | 0.000 |
| Vegetation Type + Season + Season*Vegetation Type + Temperature | 51.909 | 0.000 |
| Elevation + Season + Temperature | 60.345 | 0.000 |
| **Stage 2 Models** | **Δ AICc** | ***w*** |
| Stage 1 + Fox Probability + Coyote Probability + Bobcat Probability | 0.000 | 0.993 |
| Stage 1 + Coyote Probability + Bobcat Probability | 10.332 | 0.006 |
| Stage 1 + Fox Probability + Bobcat Probability | 13.764 | 0.001 |
| Stage 1 + Bobcat Probability | 26.660 | 0.000 |
| Stage 1 + Fox Probability + Coyote Probability | 26.917 | 0.000 |
| Stage 1 + Fox Probability | 36.679 | 0.000 |
| Stage 1 + Coyote Probability | 37.994 | 0.000 |
| Stage 1 | 49.989 | 0.000 |

*Bobcat*

| **Stage 1 Models** | **Δ AICc** | ***w*** |
| --- | --- | --- |
| Vegetation Type + Elevation + Season + Season* Vegetation Type + Temperature | 0.000 | 0.961 |
| Vegetation Type + Season + Season* Vegetation Type + Temperature | 7.359 | 0.024 |
| Vegetation Type + Canopy Cover + Understory + Season + Season* Vegetation Type + Temperature | 8.312 | 0.015 |
| Elevation + Season + Temperature | 47.932 | 0.000 |
| Elevation + Canopy Cover + Understory + Season + Temperature | 50.806 | 0.000 |
| Canopy Cover + Understory + Season + Temperature | 61.395 | 0.000 |
| **Stage 2 Models** | **Δ AICc** | ***w*** |
| Stage 1 + Skunk Probability | 0.000 | 0.473 |
| Stage 1 + Skunk Probability + Coyote Probability | 1.633 | 0.209 |
| Stage 1 + Fox Probability + Skunk Probability | 1.693 | 0.203 |
| Stage 1 + Fox Probability + Skunk Probability + Coyote Probability | 3.246 | 0.093 |
| Stage 1 | 8.342 | 0.007 |
| Stage 1 + Fox Probability | 9.060 | 0.005 |
| Stage 1 + Coyote Probability | 9.153 | 0.005 |
| Stage 1 + Fox Probability + Coyote Probability | 9.674 | 0.004 |

*Coyote*

| **Stage 1 Models** | **Δ AICc** | ***w*** |
| --- | --- | --- |
| Vegetation Type + Elevation + Season + Season*Vegetation Type + Temperature | 0.000 | 0.526 |
| Vegetation Type + Season + Season*Vegetation Type + Temperature | 1.1906 | 0.290 |
| Vegetation Type + Canopy Cover + Understory + Season + Season* Vegetation Type + Temperature | 2.098 | 0.184 |
| Elevation + Canopy Cover + Understory + Season + Temperature | 43.382 | 0.000 |
| Canopy Cover + Understory + Season + Temperature | 48.881 | 0.000 |
| Elevation + Season + Temperature | 51.617 | 0.000 |
| **Stage 2 Models** | **Δ AICc** | ***w*** |
| Stage 1A + Skunk Probability + Bobcat Probability | 0.000 | 0.149 |
| Stage 1A + Fox Probability + Skunk Probability + Bobcat Probability | 0.006 | 0.148 |
| Stage 1B + Skunk Probability + Bobcat Probability | 0.620 | 0.110 |
| Stage 1A | 0.873 | 0.097 |
| Stage 1A + Skunk Probability | 1.295 | 0.078 |
| Stage 1A + Fox Probability + Bobcat Probability | 1.465 | 0.072 |
| Stage 1A + Fox Probability | 1.573 | 0.068 |
| Stage 1A + Bobcat Probability | 1.601 | 0.067 |
| Stage 1B + Fox Probability + Skunk Probability + Bobcat Probability | 1.769 | 0.062 |
| Stage 1A + Fox Probability + Skunk Probability | 2.315 | 0.047 |
| Stage 1B + Bobcat Probability | 2.387 | 0.045 |
| Stage 1B + Fox Probability + Bobcat Probability | 3.468 | 0.026 |
| Stage 1B | 4.653 | 0.015 |
| Stage 1B + Skunk Probability | 5.976 | 0.008 |
| Stage 1B + Fox Probability | 6.650 | 0.005 |
| Stage 1B + Fox Probability + Skunk Probability | 7.991 | 0.003 |
