# Supplementary material for: Occurrence of mesocarnivores in montane sky islands: How spatial and temporal overlap informs rabies management in a regional hotspot
Source: PLoS One. 2021 Nov 5;16(11):e0259260. doi: 10.1371/journal.pone.0259260 (PMC8570508; doi:10.1371/journal.pone.0259260)
Supplement: S2 Table — (DOCX) [file pone.0259260.s002.docx]

**S2 Table. Best fit models.** Model outputs for best fit models for each mesocarnivore. Stage 1 = single-species occurrence, Stage 2 = multi-species occurrence. * Indicates a statistically significant estimate (P < 0.1).

*Gray Fox*

| **Stage 1** | | **Stage 2** | |
| --- | --- | --- | --- |
| **Variable** | **β ± SE** | **Variable** | **β ± SE** |
| Elevation | -0.282 ± 0.018* | Elevation | -0.172 ± 0.017* |
| spring 1 | -3.147 ± 0.730* | spring 1 | -0.438 ± 0.782 |
| summer 1 | 0.766 ± 0.174* | summer 1 | 1.287 ± 0.296* |
| autumn | 0.775 ± 0.183* | autumn | 1.555 ± 0.315* |
| winter | 0.504 ± 0.203* | winter | 1.033 ± 0.336* |
| spring 2 | - | spring 2 | - |
| summer 2 | -0.933 ± 0.311* | summer 2 | -1.489 ± 1.047 |
| Temperature | 0.038 ± 0.013* | Temperature | 0.031 ± 0.014* |
| Canopy Cover | 0.027 ± 0.004* | Canopy Cover | 0.003 ± 0.003 |
| Understory | 0.035 ± 0.009* | Understory | 0.024 ± 0.006* |
|  |  | Skunk Probability | 5.356 ± 0.741* |
|  |  | Coyote Probability | 6.754 ± 1.256* |

*Skunk*

| **Stage 1** | | **Stage 2** | |
| --- | --- | --- | --- |
| **Variable** | **β ± SE** | **Variable** | **β ± SE** |
| Elevation | -0.085 ± 0.018* | Elevation | -0.068 ± 0.018* |
| spring 1 | 0.662 ± 0.717 | spring 1 | 2.237 ± 0.678* |
| summer 1 | 1.391 ± 0.171* | summer 1 | 1.657 ± 0.315* |
| autumn | 1.459 ± 0.180* | autumn | 1.747 ± 0.329* |
| winter | 1.496 ± 0.199* | winter | 1.791 ± 0.343* |
| spring 2 | - | spring 2 | - |
| summer 2 | 0.581 ± 0.305 | summer 2 | 1.177 ± 0.374* |
| Temperature | 0.102 ± 0.013* | Temperature | 0.078 ± 0.012* |
| Canopy Cover | 0.023 ± 0.003* | Canopy Cover | 0.019 ± 0.003* |
| Understory | 0.023 ± 0.009* | Understory | 0.012 ± 0.007 |
|  |  | Fox Probability | 2.642 ± 0.494* |
|  |  | Bobcat Probability | 16.095 ± 1.979* |
|  |  | Coyote Probability | -4.557 ± 2.053* |

*Bobcat*

| **Stage 1** | | **Stage 2** | |
| --- | --- | --- | --- |
| **Variable** | **β ± SE** | **Variable** | **β ± SE** |
| pine-oak-juniper woodlands | 9.474 ± 9.483 | pine-oak-juniper woodlands | 9.385 ± 94.947 |
| ponderosa pine forest | 9.127 ± 9.451 | ponderosa pine forest | 0.393 ± 251.030 |
| upper evergreen forest | 9.966 ± 9.415 | upper evergreen forest | - |
| upper pine-oak woodlands | - | upper pine-oak woodlands | - |
| Elevation | 0.148 ± 0.048* | Elevation | 0.143 ± 0.005* |
| spring 1 | -1.743 ± 14.126 | spring 1 | -0.957 ± 513.620 |
| summer 1 | 0.844 ± 0.749 | summer 1 | 10.931 ± 94.942 |
| autumn | 0.418 ± 0.777 | autumn | 11.179 ± 94.942 |
| winter | 0.061 ± 0.878 | winter | 11.071 ± 94.943 |
| spring 2 | - | spring 2 | - |
| summer 2 | -13.589 ± 1.519* | summer 2 | 0.109 ± 203.300 |
| spring 1* pine-oak-juniper woodlands | -12.9.902 ± 14.554 | spring 1* pine-oak-juniper woodlands | -8.494 ± 670.220 |
| spring 1* ponderosa pine forest | -11.070 ± 14.657 | spring 1* ponderosa pine forest | 1.393 ± 719.490 |
| spring 1* upper evergreen forest | -13.566 ± 14.682 | spring 1* upper evergreen forest | - |
| spring 1* upper pine-oak woodlands | - | spring 1* upper pine-oak woodlands | - |
| summer 1* pine-oak-juniper woodlands | -0.986 ± 1.433 | summer 1* pine-oak-juniper woodlands | -10.007 ± 94.950 |
| summer 1* ponderosa pine forest | 0.605 ± 1.111 | summer 1* ponderosa pine forest | -1.120 ± 251.030 |
| summer 1* upper evergreen forest | - | summer 1* upper evergreen forest | - |
| summer 1* upper pine-oak woodlands | - | summer 1* pine-oak woodlands | - |
| autumn* pine-oak-juniper woodlands | 0.173 ± 1.408 | autumn* pine-oak-juniper woodlands | -19.856 ± 173.230 |
| autumn* ponderosa pine forest | 0.855 ± 1.078 | autumn* ponderosa pine forest | -1.046 ± 251.030 |
| autumn* upper evergreen forest | - | autumn* upper evergreen forest | - |
| winter* pine-oak-juniper woodlands | -0.583 ± 1.481 | winter* pine-oak-juniper woodlands | -19.953 ±152.68 |
| winter* ponderosa pine forest | 1.212 ± 1.151 | winter* ponderosa pine forest | -10.691 ±270.650 |
| winter* upper evergreen forest | - | winter upper evergreen forest | - |
| spring 2* pine-oak-woodlands | - | spring 2* pine-oak-woodlands | - |
| spring 2* ponderosa pine forest | - | spring 2* ponderosa pine forest | - |
| spring 2* upper evergreen forest | - | spring 2* upper evergreen forest | - |
| summer 2* pine-oak-juniper woodlands | 14.912 ± 2.666* | summer 2* pine-oak-juniper woodlands | - |
| summer 2* ponderosa pine forest | 14.835 ± 1.981* | summer 2* ponderosa pine forest | -0.276 ± 407.380 |
| summer 2* upper evergreen forest | - | summer 2* upper evergreen forest | - |
| Temperature | 0.014 ± 0.038 | Temperature | 0.026 ± 0.032 |
|  |  | Skunk Probability | 6.098 ± 1.598 |

*Coyote*

| **Stage 1** | | **Stage 2** | |
| --- | --- | --- | --- |
| **Variable** | **β ± SE** | **Variable** | **β ± SE** |
| pine-oak-juniper woodlands | 10.933 ± 3.653* | pine-oak-juniper woodlands | - |
| ponderosa pine forest | 12.035 ± 3.636* | ponderosa pine forest | - |
| upper evergreen forest | 11.073 ± 3.605* | upper evergreen forest | - |
| upper pine-oak woodlands | - | upper pine-oak woodlands | - |
| Elevation | -0.122 ± 0.068 | Elevation | -0.086 ± 0.070 |
| spring 1 | 0.799 ± 5.411 | spring 1 | 3.139 ± 1.2884* |
| summer 1 | 0.803 ± 0.378* | summer 1 | 1.766 ± 0.749* |
| autumn | 1.036 ± 0.402* | autumn | 1.761 ± 0.019* |
| winter | 0.598 ± 0.451 | winter | 1.593 ± 0.042 |
| spring 2 | - | spring 2 | - |
| summer 2 | -11.426 ± 0.949* | summer 2 | -11.340 ± 514.940 |
| spring 1* pine-oak-juniper woodlands | 0.300 ± 5.617 | spring 1* pine-oak-juniper woodlands | -1.217 ± 1.468 |
| spring 1* upper evergreen forest | 0.875 ± 5.710 | spring 1* upper evergreen forest | - |
| spring 1* upper pine-oak woodlands | - | spring 1* upper pine-oak woodlands | - |
| summer 1* pine-oak-juniper woodlands | -0.362 ± 0.762 | summer 1* pine-oak-juniper woodlands | -1.042 ± 0.737 |
| summer 1* ponderosa pine forest | -1.952 ± 0.629* | summer 1* ponderosa pine forest | -0.894 ± 0.511* |
| summer 1* upper evergreen forest | - | summer 1* upper evergreen forest | - |
| summer 1* upper pine-oak woodlands | - | summer 1* upper pine-oak woodlands | - |
| autumn* pine-oak-juniper woodlands | -2.990 ± 0.759* | autumn* pine-oak-juniper woodlands | -1.986 ± 1.015* |
| autumn* ponderosa pine forest | -1.5233 ± 0.628* | autumn* ponderosa pine forest | -0.460 ± 0.346 |
| autumn* upper evergreen forest | - | autumn* upper evergreen forest | - |
| winter* pine-oak-juniper woodlands | -0.131 ± 0.805 | winter* pine-oak-juniper woodlands | 0.112 ± 0.486 |
| winter* ponderosa pine forest | -0.304 ± 0.662 | winter* ponderosa pine forest | 0.952 ± 0.337* |
| winter* upper evergreen forest | - | winter* upper evergreen forest | - |
| spring 2* pine-oak-juniper woodlands | - | spring 2* pine-oak-juniper woodlands | 1.693 ± 0.905* |
| spring 2* ponderosa pine forest | - | spring 2* ponderosa pine forest | 2.033 ± 0.830* |
| spring 2* upper evergreen forest | - | spring 2* upper evergreen forest | - |
| summer 2* ponderosa pine forest | - | summer 2* ponderosa pine forest | - |
| Temperature | -0.001 ± 0.021 | Temperature | -0.027 ± 0.019 |
|  |  | Skunk Probability | -2.653 ± 1.827 |
|  |  | Bobcat Probability | 13.716 ± 6.897* |
