# Supplementary material for: Occurrence of mesocarnivores in montane sky islands: How spatial and temporal overlap informs rabies management in a regional hotspot
Source: PLoS One. 2021 Nov 5;16(11):e0259260. doi: 10.1371/journal.pone.0259260 (PMC8570508; doi:10.1371/journal.pone.0259260)
Supplement: S3 Table — (DOCX) [file pone.0259260.s003.docx]

**S3 Table. Type 3 Test.** Type 3 test of fixed effects for two stage modeling process for each mesocarnivore. Stage 1 = single-species occurrence, Stage 2 = multi-species occurrence. A = top model, B–I = competing models.

*Gray Fox*

| **Stage 1** | | | |
| --- | --- | --- | --- |
| **Effect** | **Number DF** | **F Value** | **Pr > F** |
| Elevation | 1 | 246.10 | < 0.001 |
| Canopy Cover | 1 | 79.21 | < 0.001 |
| Understory | 1 | 16.00 | < 0.001 |
| Season | 5 | 14.91 | < 0.001 |
| Temperature | 1 | 8.28 | 0.0040 |
| **Stage 2A** | | | |
| **Effect** | **Number DF** | **F Value** | **Pr > F** |
| Elevation | 1 | 107.80 | < 0.001 |
| Canopy Cover | 1 | 0.58 | 0.4478 |
| Understory | 1 | 13.98 | 0.0002 |
| Season | 5 | 8.50 | < 0.001 |
| Temperature | 1 | 5.14 | 0.0234 |
| Skunk Probability | 1 | 52.63 | < 0.001 |
| Coyote Probability | 1 | 28.91 | < 0.001 |
| **Stage 2B** | | | |
| **Effect** | **Number DF** | **F Value** | **Pr > F** |
| Elevation | 1 | 75.96 | < 0.001 |
| Canopy Cover | 1 | 0.43 | 0.5122 |
| Understory | 1 | 14.46 | 0.0001 |
| Season | 5 | 8.67 | < 0.001 |
| Temperature | 1 | 5.00 | 0.0254 |
| Skunk Probability | 1 | 48.67 | < 0.001 |
| Bobcat Probability | 1 | 30.26 | < 0.001 |
| Coyote Probability | 1 | 1.22 | 0.2694 |

*Skunk*

| **Stage 1** | | | |
| --- | --- | --- | --- |
| **Effect** | **Number DF** | **F Value** | **Pr > F** |
| Elevation | 1 | 23.15 | < 0.001 |
| Canopy Cover | 1 | 62.02 | < 0.001 |
| Understory | 1 | 6.94 | 0.0084 |
| Season | 5 | 19.58 | < 0.001 |
| Temperature | 1 | 63.35 | < 0.001 |
| **Stage 2A** | | | |
| **Effect** | **Number DF** | **F Value** | **Pr > F** |
| Elevation | 1 | 14.83 | 0.0001 |
| Canopy Cover | 1 | 37.71 | < 0.001 |
| Understory | 1 | 2.44 | 0.1182 |
| Season | 5 | 6.91 | < 0.001 |
| Temperature | 1 | 39.07 | < 0.001 |
| Fox Probability | 1 | 28.65 | < 0.001 |
| Bobcat Probability | 1 | 66.14 | < 0.001 |
| Coyote Probability | 1 | 4.93 | 0.0265 |

*Bobcat*

| **Stage 1** | | | |
| --- | --- | --- | --- |
| **Effect** | **Number DF** | **F Value** | **Pr > F** |
| Elevation | 1 | 9.37 | 0.0022 |
| Vegetation Type | 3 | 2.24 | 0.0814 |
| Season | 5 | 10.05 | < 0.001 |
| Season * Vegetation Type | 11 | 6.63 | < 0.001 |
| Temperature | 1 | 0.14 | 0.7112 |
| **Stage 2A** | | | |
| **Effect** | **Number DF** | **F Value** | **Pr > F** |
| Elevation | 1 | 7.96 | 0.0048 |
| Vegetation Type | 2 | 0.00 | 0.9999 |
| Season | 5 | 0.01 | 1.0000 |
| Season * Vegetation Type | 9 | 0.00 | 1.0000 |
| Temperature | 1 | 0.66 | 0.4179 |
| Skunk Probability | 1 | 14.57 | 0.0001 |
| **Stage 2B** | | | |
| **Effect** | **Number DF** | **F Value** | **Pr > F** |
| Elevation | 1 | 6.83 | 0.0090 |
| Vegetation Type | 2 | 0.00 | 0.9999 |
| Season | 5 | 0.00 | 1.0000 |
| Season * Vegetation Type | 9 | 0.00 | 1.0000 |
| Temperature | 1 | 0.63 | 0.4289 |
| Skunk Probability | 1 | 13.87 | 0.0002 |
| Coyote Probability | 1 | 0.11 | 0.7386 |
| **Stage 2C** | | | |
| **Effect** | **Number DF** | **F Value** | **Pr > F** |
| Elevation | 1 | 8.51 | 0.0035 |
| Vegetation Type | 2 | 0.00 | 0.9999 |
| Season | 5 | 0.00 | 1.0000 |
| Season * Vegetation Type | 9 | 0.00 | 1.0000 |
| Temperature | 1 | 0.72 | 0.3958 |
| Fox Probability | 1 | 13.02 | 0.0003 |
| Skunk Probability | 1 | 0.73 | 0.3921 |

*Coyote*

| **Stage 1A** | | | |
| --- | --- | --- | --- |
| **Effect** | **Number DF** | **F Value** | **Pr > F** |
| Elevation | 1 | 3.20 | 0.0736 |
| Vegetation Type | 3 | 7.51 | < 0.001 |
| Season | 5 | 32.45 | < 0.001 |
| Season * Vegetation Type | 8 | 4.45 | < 0.001 |
| Temperature | 1 | 0.00 | 0.9784 |
| **Stage 1B** | | | |
| **Effect** | **Number DF** | **F Value** | **Pr > F** |
| Vegetation Type | 3 | 7.77 | < 0.001 |
| Season | 5 | 32.54 | < 0.001 |
| Season * Vegetation Type | 8 | 4.41 | < 0.001 |
| Temperature | 1 | 0.00 | 0.9820 |
| **Stage 2A** | | | |
| **Effect** | **Number DF** | **F Value** | **Pr > F** |
| Elevation | 1 | 1.48 | 0.2237 |
| Vegetation Type | 2 | 2.15 | 0.1162 |
| Season | 5 | 2.39 | 0.0355 |
| Season * Vegetation Type | 7 | 3.22 | 0.0021 |
| Temperature | 1 | 2.11 | 0.1460 |
| Skunk Probability | 1 | 2.11 | 0.1465 |
| Bobcat Probability | 1 | 3.96 | 0.0468 |
| **Stage 2B** | | | |
| **Effect** | **Number DF** | **F Value** | **Pr > F** |
| Elevation | 1 | 1.59 | 0.2079 |
| Vegetation Type | 2 | 2.05 | 0.1295 |
| Season | 5 | 2.40 | 0.0349 |
| Season * Vegetation Type | 7 | 3.24 | 0.0020 |
| Temperature | 1 | 2.12 | 0.1452 |
| Fox Probability | 1 | 0.12 | 0.7303 |
| Skunk Probability | 1 | 2.09 | 0.1478 |
| Bobcat Probability | 1 | 4.08 | 0.0434 |
| **Stage 2C** | | | |
| **Effect** | **Number DF** | **F Value** | **Pr > F** |
| Vegetation Type | 2 | 2.72 | 0.0661 |
| Season | 5 | 2.45 | 0.0318 |
| Season * Vegetation Type | 7 | 3.20 | 0.0022 |
| Temperature | 1 | 2.06 | 0.1513 |
| Skunk Probability | 1 | 2.13 | 0.1447 |
| Bobcat Probability | 1 | 8.11 | 0.0044 |
| **Stage 2D** | | | |
| **Effect** | **Number DF** | **F Value** | **Pr > F** |
| Elevation | 1 | 4.13 | 0.0423 |
| Vegetation Type | 2 | 3.99 | 0.0186 |
| Season | 5 | 2.31 | 0.0414 |
| Season * Vegetation Type | 7 | 3.39 | 0.0013 |
| Temperature | 1 | 2.15 | 0.1422 |
| **Stage 2E** | | | |
| **Effect** | **Number DF** | **F Value** | **Pr > F** |
| Elevation | 1 | 4.99 | 0.0256 |
| Vegetation Type | 2 | 2.76 | 0.0635 |
| Season | 5 | 2.14 | 0.0579 |
| Season * Vegetation Type | 7 | 3.42 | 0.0012 |
| Temperature | 1 | 2.22 | 0.1365 |
| Skunk Probability | 1 | 0.42 | 0.5149 |
| **Stage 2F** | | | |
| **Effect** | **Number DF** | **F Value** | **Pr > F** |
| Elevation | 1 | 1.62 | 0.2031 |
| Vegetation Type | 2 | 2.98 | 0.0506 |
| Season | 5 | 2.24 | 0.0478 |
| Season * Vegetation Type | 7 | 3.11 | 0.0028 |
| Temperature | 1 | 2.17 | 0.1404 |
| Fox Probability | 1 | 0.13 | 0.7143 |
| Bobcat Probability | 1 | 2.06 | 0.1516 |
| **Stage 2G** | | | |
| **Effect** | **Number DF** | **F Value** | **Pr > F** |
| Elevation | 1 | 3.66 | 0.0557 |
| Vegetation Type | 2 | 3.15 | 0.0429 |
| Season | 5 | 2.12 | 0.0599 |
| Season * Vegetation Type | 7 | 3.34 | 0.0015 |
| Temperature | 1 | 2.23 | 0.1354 |
| Fox Probability | 1 | 0.00 | 0.9879 |
| **Stage 2H** | | | |
| **Effect** | **Number DF** | **F Value** | **Pr > F** |
| Elevation | 1 | 1.50 | 0.2211 |
| Vegetation Type | 2 | 3.16 | 0.0426 |
| Season | 5 | 2.23 | 0.0486 |
| Season * Vegetation Type | 7 | 3.09 | 0.0030 |
| Temperature | 1 | 2.17 | 0.1411 |
| Bobcat Probability | 1 | 1.92 | 0.1663 |
| **Stage 2I** | | | |
| **Effect** | **Number DF** | **F Value** | **Pr > F** |
| Vegetation Type | 2 | 2.72 | 0.0661 |
| Season | 5 | 2.45 | 0.0319 |
| Season * Vegetation Type | 7 | 3.18 | 0.0023 |
| Temperature | 1 | 2.06 | 0.1514 |
| Fox Probability | 1 | 0.00 | 0.9574 |
| Skunk Probability | 1 | 2.13 | 0.1447 |
| Bobcat Probability | 1 | 6.88 | 0.0087 |
